# Creation of a Métis-Specific Instrument for Cancer Screening: A Scoping Review of Cancer-Screening Programs and Instruments

**DOI:** 10.3390/curroncol30110715

**Published:** 2023-11-09

**Authors:** Angeline Letendre, Momtafin Khan, Reagan Bartel, Bonnie Chiang, Ashton James, Brittany Shewchuk, June Kima, Meghan Macphail, Marcus Vaska, Monica Schwann, Huiming Yang, Karen A. Kopciuk

**Affiliations:** 1 Cancer Prevention & Screening Innovation, Provincial, Population and Public Health, Alberta Health Services, Edmonton, AB T5J 3E4, Canada; angeline.letendre@albertahealthservices.ca; 2 Cancer Epidemiology and Prevention Research, Cancer Care Alberta, Alberta Health Services, Calgary, AB T2S 3C3, Canada; momtafin.khan@albertahealthservices.ca (M.K.);; 3 Métis Nation of Alberta, Edmonton, AB T5G 0X5, Canada; health@metis.org (R.B. & A.J. & J.K.); 4 Screening Programs, Alberta Health Services, Calgary, AB T2S 3C3, Canada; bonnie.chiang@albertahealthservices.ca (B.C.); monica.schwann@albertahealthservices.ca (M.S.); huiming.yang@albertahealthservices.ca (H.Y.); 5 Department of Community Health Sciences, University of Calgary, Calgary, AB T2N 4Z6, Canada; meghan.macphail1@ucalgary.ca; 6 Knowledge Resource Service, Alberta Health Services, Calgary, AB T2N 4N2, Canada; marcus.vaska@albertahealthservices.ca; 7 Departments of Oncology and Mathematics and Statistics, University of Calgary, Calgary, AB T2N 1N4, Canada

**Keywords:** barriers and facilitators, Canadian indigenous study, breast cancer screening, cervical cancer screening, colorectal cancer screening, Métis people, Métis survey

## Abstract

Understanding the barriers to and facilitators of cancer screening programs among Indigenous populations remains limited. In the spirit of mutual respect, this co-led, collaborative project was carried out between the Métis Nation of Alberta and Screening Programs from Alberta Health Services (AHS). This scoping review assessed the cancer screening literature for available questionnaires and then identified themes and suitable questions for a Métis-specific cancer screening questionnaire. Literature searches on cervical, breast, and colorectal cancer screening programs and related concepts were conducted in electronic databases, including the Native Health Database, MEDLINE (Ovid), PsycINFO, PubMed, PubMed Central, CINAHL, MEDLINE (Ebsco), Psychology & Behavioral Sciences Collection, and Web of Science. Grey literature was collected from AHS Insite, Open Archives Initiative repository, American Society of Clinical Oncology, European Society of Medical Oncology, Google, and Google Scholar. 135 articles were screened based on the eligibility criteria with 114 articles selected, including 14 Indigenous-specific ones. Knowledge, attitude, belief, behaviour, barrier, and facilitator themes emerged from the review, but no Métis-specific cancer screening instruments were found. Thus, one was developed using existing cancer screening instruments, with additional questions created by the project team. A survey of the Métis population in Alberta will use this questionnaire and provide data to address the burden of cancer among Métis people.

## 1. Introduction

Despite improvements in treatments and therapies, cancer continues to be the leading cause of death in Canada. However, early detection and treatment of pre-cancerous lesions and cancer in asymptomatic people through screening can reduce the burden of cancer, including death from cancer [1,2,3]. Cancer screening programs for breast, cervical, and colorectal cancer exist in most provinces and some territories in Canada, with pilots for lung cancer recently being implemented. Organized cancer screening programs are cost-effective approaches that reduce mortality and morbidity in populations [4]. This is an important consideration for single-payer healthcare systems, such as the Canadian system.

Trends in incidence and mortality for these three cancer screening programs illustrate their impact over time following their implementation. For example, the annual percent change (APC) in age-standardized mortality rates for breast cancer in Canadian females had declines of −1.5% per year from 2010 to 2020 due to organized screening as well as improved treatments [5]. Colorectal cancer screening has substantially contributed to declines in both incidence (−3.9% per year) and mortality rates (−3.4%) in Canadian males and females from 2014 to 2020. Cervical cancer incidence and mortality rates from 1972 to2004 declined more significantly, with the use of the Papanicolaou (Pap) test being credited with early detection of pre-cancerous lesions. Most Canadians report having at least one screening test in their lifetimes [6]. In 2017, 91.4% of Canadian women aged 50 to 74 years reported having at least one mammogram in their lifetime while, in the same year, 90.6% of Canadian women aged 25 to 69 years old reported that they had at least one Pap smear in their lifetime [1]. Among Canadians aged 50–74 years old, 60.9% reported having a fecal occult blood test (FOBT) in their lifetime (females—62.3%; males—59.4%) [1].

While these statistics represent Canada overall, distinct ethnographic groups, including Indigenous people, have their own ways of life that are grounded in their cultures, worldviews, and experiences. Métis people in Canada have been greatly impacted by colonization and historical trauma, including residential schooling, the Sixties Scoop, influence from federal and provincial legislation, and ongoing racism and discrimination. These factors have been shown to contribute to negative outcomes, such as increased risks for poverty, and substance use including alcohol and drug addiction, sexual abuse, homelessness, and high rates of suicide, depression, and chronic disease. All of these circumstances lead to poor health outcomes and pose factors that result in inequities for both the burden of cancer and the use of cancer-related screening and treatment services among Indigenous people. Further complicating these issues is the propensity for research in Canada to focus on a pan-Indigenous approach to data that ignores the specific contexts in which First Nations, Métis, and Inuit people live. These contexts, including diversity in culture, knowledge and beliefs, and language, may also lead to variation in the barriers and facilitators for participation in cancer screening and the subsequent increased incidence of some cancers [7]. Current research on barriers to and facilitators of cancer screening among Métis people is limited, despite being a population that makes up at least a third of the entire Canadian Indigenous population [8].

### Who Are the Métis

The Métis are Indigenous people of mixed European and First Nation descent with their own cultures, knowledge systems, and language (Michif). They are distinct from other constitutionally recognized Indigenous peoples in Canada (First Nations, Inuit) [9,10]. Métis people reside in traditional and land-based communities, as well as urban settings across Canada, including Alberta, which is home to the only Métis Settlements in Canada. In the 2021 Census, 127,475 people in Alberta self-identified as Métis [11]. More than 5630 Métis people are a registered member of one of eight Métis Settlements across Northern Alberta, and more than 63,000 Métis people are a registered citizen of the Métis Nation of Alberta [12]. A 2021 scoping review suggests that Métis people have reduced participation rates in cancer screening (specifically cervical, breast, and colorectal cancer) and increased cancer incidence compared to the overall Canadian averages and non-Indigenous Canadians [13]. Another study based on 2007 to 2011 combined data from the annual Canadian Community Health Survey found that, among Métis people living in Ontario, cancer participation rates were lower compared to non-Indigenous (defined as not off reserve First Nations or Métis) people [14]. Screening uptake prevalence for cervical and breast cancer were 72% and 59%, respectively, for Métis females compared to 78% and 68%, respectively, for non-Indigenous females. Screening uptake for colorectal cancer screening was substantially lower for both population groups compared to the other two screening programs. For males, the prevalence of an FOBT (Fecal Occult Blood Test) in the past 2 years was 23.6% for Métis males compared to 25.5% for non-Indigenous males. For females, the prevalence was 22.4% for Métis females compared to 28.2% for non-Indigenous females [14]. An Alberta study reported that Métis people were 86% less likely to participate in colorectal screening in comparison to other ethnic groups (First Nation, immigrant, Chinese and Black minorities) [15]. The most recent Canada-wide study, conducted by Mazereeuw et al. [16], also found Métis people were less likely to participate in breast, cervical, and colorectal screening when compared to non-Indigenous people. However, a narrative review of relevant research on Canadian Indigenous populations, including Métis populations, revealed a lack of literature on cervical, breast, and colorectal cancer screening. Thus, a significant research gap exists on the cancer screening rates, behaviours, barriers and facilitators amongst the Métis population [17].

Overall, there is limited research on cancer screening barriers, facilitators, beliefs, attitudes, knowledge, and behaviours of Alberta’s Métis population, preventing the development of culturally appropriate programs to address cancer risk factors and barriers to care. The strategy for this review was created in close collaboration between the Métis Nation of Alberta, including Indigenous researchers with expertise in Indigenous health, health research and cancer research with Indigenous people, and Screening Programs from Alberta Health Services. The purpose of this scoping review was to review the current literature on cancer screening questionnaires and to utilize this information to create a Métis-specific questionnaire to assess these cancer-screening themes. 

## 2. Materials and Methods

The research team adhered to the ethical principles and practices in alignment with the Tri-Council Policy Statement: 2 (2022)–Chapter 9: Research Involving the First Nations, Inuit and Métis Peoples of Canada; Principles of Ethical Métis Research; and First Nations Principles of OCAP^®^ (ownership, control, access, and possession) in carrying out this research project [18,19,20]. By working together with Métis researchers and health leaders, the research team benefitted from incorporating Métis perspectives and traditional knowledge throughout this project. This collaboration ensured that the Métis communities will benefit from this research and the resulting knowledge of their data. 

A scoping review of the existing literature on cancer screening questionnaires for cervical, breast, and colorectal cancer was performed. The identified articles were then characterized and summarized to create a Métis-specific survey on cancer screening practices as well as barriers to and facilitators of cancer screening. 

### 2.1. Data Sources

A comprehensive search of the relevant literature was conducted in the electronic databases The Native Health Database, MEDLINE (Ovid), PsycINFO, PubMed, PubMed Central, CINAHL, MEDLINE (Ebsco), Psychology & Behavioral Sciences Collection, and Web of Science. Additionally, grey literature was collected from Alberta Health Services (AHS) Insite, OAIster (Open Archives Initiative repository), ASCO (American Society of Clinical Oncology), ESMO (European Society of Medical Oncology), Google, and Google Scholar. 

### 2.2. Search Strategy

Searches were conducted between 7 June 2021 and 9 June 2021. Papers were not excluded based on their publication date, thus any citations from database inception until 9 June 2021 (date of search completion) were eligible for inclusion. Search terms were informed by the study’s research objectives. Each concept and its synonyms were included as Medical Subject Headings (MeSH) and keyword search terms and are listed in Table 1. Cancer search filters and the Indigenous search filter were also utilized, though they are not listed in the table. “Indigenous Peoples” is an overarching MeSH term that also captures specific terms such as American Indian or Alaska Native, Australian, Aboriginal and Torres Strait Islander Peoples, Maori People, and Native Hawaiian or Other Pacific Islander in addition to Indigenous Peoples. 

The Boolean operator “AND” was used to join major topics and “OR” to join keywords within the same concept category. Questionnaire and Breast/Cervical/Colorectal Cancer Screening keywords were always included and combined with other concepts in the search strings. MeSH headings were used to replace keywords if available in each database. 

### 2.3. Study Selection 

**Figure 1:**
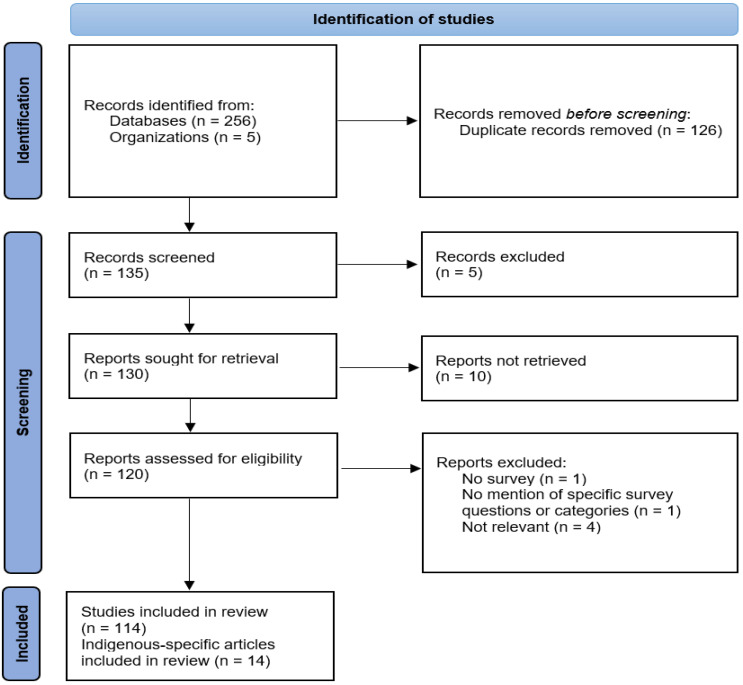
PRISMA flow diagram of the article screening process.

After removing duplicates, 135 citations were identified by the search strategy. Following title and abstract screening of all 135 records between 9 June and 2 August 2021, 120 were selected for full-text review (Figure 1). Finally, 114 articles were eligible for inclusion in the scoping review. The PRISMA flow diagram (Figure 1) presents a summary of the review and inclusion/exclusion process. Papers were excluded if they did not use a questionnaire instrument, did not mention specific questionnaire questions or categories, or were not relevant to the goals of this scoping review. 

### 2.4. Data Collection

For each publication, information was gathered on (1) the cancer screening questionnaire used in the project, (2) the validity of the cancer screening questionnaire, (3) the population for which the questionnaire was created including Indigenous populations, and (4) the relevant cancer-screening related findings from each study. 

### 2.5. Data Charting

After the data collection was organized, a thematic analysis was performed. Initially, a spreadsheet summarized the study’s information such as author, title, year of publication, sample size, population, research type, the type of cancer studied (breast, cervical, and/or colorectal), and cancer prevalence and screening rates in the target population. Six thematic umbrella terms were developed based on the study objectives and included attitudes, beliefs, knowledge, barriers, facilitators, and behaviors. Sub-themes within each umbrella term were identified in each study and recorded using consistent wording to easily identify emerging patterns and most common themes. Of the 114 included articles, those studying Indigenous populations were targeted for further data extraction and analysis, with a focus on the survey instrument used—specifically, whether it was validated and included in the publication. These would provide further insight into useful and appropriate questions being asked to Indigenous populations and their relevancy in characterizing screening program concepts. 

## 3. Results 

### 3.1. Themes across the Included Articles 

In the 114 articles reviewed, the most common sub-themes under each of the six umbrella terms were identified with a full-text review. Initially, general sub-themes were identified as they emerged from the text, then categorized into one of the six umbrella terms. These sub-themes were given short, descriptive names so that as more articles were reviewed and certain themes appeared more often, the data extraction process remained consistent. Table 2 describes the umbrella terms and the most common sub-themes. 

Within the umbrella term of *attitudes*, articles included participants’ self-efficacy in their ability to live a healthy lifestyle and whether they were motivated to take care of their own health, including by getting regular check-ups and asymptomatic screenings. Under the umbrella term *knowledge*, the most common sub-themes included whether participants had an awareness of cancer screening practices, the purpose of cancer-screening, cancer risk factors, and the likelihood of cure. In cancer-screening *behaviours*, studies inquired after participants’ screening histories and compliance with physician recommendations to be screened. Under *beliefs*, studies discussed perceived barriers, benefits, and risks to cancer screening, perceived susceptibility to cancer, as well as participants’ belief in the severity of the disease. Common *barriers* included embarrassment around the screening process (e.g., removing clothing), lack of health insurance or high coinsurance costs, language barriers, lack of transportation, and worry about the ramifications of receiving a positive screening result. However, there was a lack of discussion of sub-themes under *facilitators* to screening. Some notable facilitators included prior screening, a good experience with their physician, social support, and provider recommendations for cancer screening.

### 3.2. Creation of a Métis-Specific Cancer-Screening Instrument

Very limited Métis-specific research on cancer-screening topics has been conducted and no Métis-specific cancer-screening instruments were found in our scoping review. Based on the thematic analysis of the 114 articles included in the scoping review, the team collectively decided to develop questions on barriers to and facilitators of cancer screening. The project team then concentrated on an in-depth review of the 14 Indigenous population-specific articles included in this scoping review; however, none were found to be particularly relevant to the topics of facilitators of and barriers to cancer screening. A summary table of these 14 articles is included in the Supplementary Materials (Table S1). Twelve of these Indigenous population-specific articles were based on U.S. studies involving various Native American tribes with the remaining two based on Inuit and First Nations communities in Canada. Most focused on a single cancer screening program although three articles considered breast, cervical, and colorectal cancer screening.

Cancer screening-focused questions on behaviours, beliefs and knowledge were selected or adapted from the following pre-existing surveys: the *Canadian Community Heath Survey* 2020 (CCHS) [21], the *Cervical Cancer Knowledge Prevention-64* (CCKP-64) [22] and the *Breast Cancer Screening Beliefs Questionnaire* (BCSBQ) [23]. Additional demographic questions were created by the Métis Nation of Alberta (MNA) that were included in the cancer-screening instrument. The survey avoided binary gender descriptors that were violently imposed through colonialism and the residential school system [24,25]. Rather, it considered other inclusive and culturally relevant choices (e.g., two-spirited) to describe self-identified gender of Métis respondents. The cancer-screening instrument was edited in multiple iterations amongst the team members until a consensus was reached on the final survey. This questionnaire was pilot tested with staff from the MNA and Screening Programs to ensure face and content validity and to identify any issues with comprehension. The final version of the questionnaire (see File S1) was programmed in Research Electronic Data Capture (REDCap, v.7) at the University of Calgary for the survey launch. The MNA facilitated telephone completion of the survey in English or in Cree for Métis Albertans who could experience barriers to survey completion related to an ability to read or write English. Consent forms were created and included in the anonymous survey that was coded in REDCap so that Métis people could participate in this Métis-specific cancer-screening survey. 

## 4. Discussion 

The scoping review found common themes across cancer screening articles including cancer screening behaviours, attitudes including self-efficacy to be healthy, beliefs on perceived barriers, benefits, and risks related to cancer screening, and barriers to accessing or performing cancer screening. However, few articles discussed facilitators to participation in their cancer screening program or survey. Amongst the 114 relevant articles, few screening instruments are adapted to minority populations. Although some instruments were adapted to minority populations to address cultural and language barriers, such as instruments translated to Chinese for Chinese populations [26,27] and an instrument adapted to address Korean culture [28], similar instruments were not available for Indigenous or Métis populations. Based on this scoping review, the newly developed questionnaire included a section on barriers and facilitators likely relevant to the Métis population. The questionnaire has been used in a survey conducted in the fall of 2022 to evaluate cancer screening rates and barriers and facilitators to cancer screening among Métis Albertans. 

While none of the identified articles were Métis-specific, important barriers were found that could be relevant for this population. One of the major barriers found across most articles was a lack of education or awareness of cancer screening. Two studies noted that the method of learning about cancer and cancer screening for participants was from media sources rather than physicians [29,30]. This suggests a lack of recommendations and knowledge translation from physicians to patients on cancer screening guidelines [31,32]. This is important to the Inuit, who tend to only distribute information of which they are absolutely certain. Therefore, they have an overall low level of awareness of HPV and its risk for cervical cancer, as any Inuit individuals who may be aware may not share the information. This barrier contributes to a lack of knowledge and, in turn, reduced cancer screening in this group [30]. Furthermore, Indigenous populations emphasize oratory methods of learning. It was found that in the Inuit population, oratory types of knowledge distribution, such as radio programs and conversations with a healthcare provider, were crucial for increasing health knowledge in this population [30]. One study found that women who were aware of preventative health behaviours, such as cancer screening, were more motivated to do so [33]. Lack of education and awareness may contribute to some of the barriers found in this review, including embarrassment and worry, in conducting cancer screening. While addressing this barrier through increased education, researchers must be culturally sensitive and be able to skillfully incorporate cultural components into their delivery of information [27,34,35]. This is especially true for Indigenous populations [35]. 

Very few of the studies in this review investigated facilitators of cancer screening, however, those that did found interesting results. For example, physician recommendations can improve the individuals’ self-efficacy and rates of cancer screening [32,36]. Multiple studies suggest that culturally appropriate programs and culturally aware professionals can improve health outcomes and improve screening rates [27,34,37,38].Findings from a cancer screening survey of Northern Plains Indian Reservation females suggested that women may participate more in cancer screening after educational sessions and discussions with clinicians about mammography [39]. This finding may be applicable to other Indigenous groups, such as Métis people, if the educational content is culturally appropriate. Another validated Indigenous-specific study of Hopi Women was focused on cancer screening programs rather than questionnaires [40]. Although the cancer screening program primarily addressed barriers in the community, the researchers did not discuss facilitators of the cancer screening program itself [40]. Furthermore, each Indigenous group has their own unique cultures and ways of life, so while the results for the Hopi women cancer screening program are validated, they may not be generalizable to other Indigenous groups with their own unique set of barriers and facilitators [40,41]. 

While specific cancer screening recommendations for Métis populations have not been discussed in the research literature, it may be useful to adapt the recommendations for cancer survivorship to screening in Indigenous people. To facilitate knowledge translation and the use of cancer screening in Indigenous populations, approaches should be specifically tailored to their needs, preferences, and barriers that stem from their unique histories and cultures [42,43]. Although a survey for First Nations peoples [44] has been created that includes cancer screening questions, none are tailored to Métis peoples. Additionally, the First Nations survey does not assess cancer screening behaviors, barriers, or facilitators to cancer screening, and thus cannot aid in the creation of tailored cancer screening programs. A recent scoping review states that there is an inadequate amount of Métis-specific cancer data (77 data records across Canada), highlighting the need for a coordinated approach to create targeted cancer prevention interventions and reduce health inequities faced by Métis people [13].

Some limitations of this scoping review are the small number of published articles about cancer screening questionnaires, with very few specific to Indigenous populations. Of the 14 articles that focused on Indigenous populations and cancer screening (Supplementary Table S1), only two were from Canada and neither included the Métis people. Another limitation is the focus on the umbrella terms of barriers and facilitators to the exclusion of attitudes, knowledge, behaviours, and beliefs. A comprehensive survey instrument could ask about these themes as well in order to more fully understand the perspectives of Indigenous people on these related topics. Lastly, the Métis-specific questionnaire may require adaptation to other Indigenous groups to capture their unique lived experiences with cancer screening. 

This scoping review highlights a need for more research on cancer screening in the Métis population and the need to not only address barriers, but also investigate and implement facilitators that are culturally appropriate. A combination of increasing facilitators and mitigating barriers identified by Métis communities may increase participation in cancer screening programs. This scoping review led to the creation of the Métis-specific cancer screening questionnaire which will provide a basis on which to build future research and to create useful tools to improve the health of the Métis people. Further investigation into the validity and usefulness of the newly created instrument is needed. In the future, cancer screening related research should increase its focus on specific Indigenous populations, including the Métis population, to promote and enable better health for all Canadians. 

## Figures and Tables

**Table 1:** Concepts, keywords and synonyms used in literature search.

Concept	Synonym (Keyword)	Synonym (MeSH)
Questionnaires	Survey*; questionnaire*; “instrument development”; psychometric*; scale*; metric*; benchmark*; matrix; measure*; indicator*	“surveys and questionnaires”; psychometrics; benchmarking
Evaluation	Knowledge; validat*; belief*; “psychometric testing”; assess*; evaluat*	Knowledge; health knowledge, attitudes, practice; validation study; culture; evaluation study
Cancer Screening Programs	“early detection of cancer”; “mass screening; “cancer prevention”; “cancer screening”; screen* adj3 cancer	“early detection of cancer”; mass screening
Cancer Types	“breast cancer”; “breast neoplasm*”; “breast carcinoma”; “breast tumor*” ’ “breast tumour*”; “cervical cancer”; “cervical neoplasm*”; “cervical carcinoma”; “cervical tumor*”; “cervical tumour*”; “colorectal cancer”; “colorectal neoplasm*”; “colorectal carcinoma”; “colorectal tumor*”; “colorectal tumour*”; “colon cancer”; “colonic neoplasm*”; “colonic carcinoma”; “colonic tumour*”; “colonic tumor*”; “colon neoplasm*”; “colon carcinoma”; “colon tumor*”; “colon tumour*”; “rectal cancer”; “rectal neoplasm*”; “rectal carcinoma”; “rectal tumor*”; “rectal tumour*”	breast neoplasms; uterine cervical neoplasms; colorectal neoplasms; colonic neoplasms; rectal neoplasms
Cancer ^1^	Cancer; neoplasm*; carcinoma; tumor*; tumour*; oncology; “medical oncology”	neoplasms; carcinoma; medical oncology
Breast/Cervix/Colon/Rectum	Breast; cervix	Breast; cervix uteri; colon; rectum
Breast/Cervical/Colorectal Cancer Screening	“breast cancer screening”; “cervical cancer screening”; “colorectal cancer screening”; screen* adj3 “breast cancer”; screen* adj3 “cervical cancer”; screen* adj3 “colorectal cancer”; mammography; mammogram*; “pap smear”; “Papanicolaou test”; “fecal immunochemical test”; “faecal immunochemical test”; “fecal occult blood test”; “faecal occult blood test”; colonoscopy	Mammography; Papanicolaou Test; occult blood; colonoscopy
Indigenous/Aboriginal ^2^	Indigenous; Aboriginal; Metis; Inuit*; “First Nations”; “native people”; “native Canadian”; Maori; “native American”	indigenous peoples; indigenous Canadians; American natives; Indians, North American; Inuits; oceanic ancestry group

* truncation symbol. ^1^ Cancer search filter consulted: Cancer-Google Docs (https://docs.google.com/document/d/1xP223elUpudJ-L7rdxxvTNbH0kQqfjn4xxxVMeRKF_c/edit?pli=1, accessed on 7 June 2023). ^2^ Indigenous search filter consulted: Indigenous Peoples-Health Sciences Search Filters-Subject Guides at University of Alberta Libraries (https://guides.library.ualberta.ca/c.php?g=734066&p=5281325, accessed on 8 June 2023).

**Table 2:** Umbrella terms and their top sub-themes identified in literature review.

Umbrella Terms	Top Sub-Themes
Attitudes	Health motivation, general health motivation, general health checkup, asymptomatic screening, self-efficacy
Knowledge	Awareness of cure, purpose of screening, awareness of risk factors, awareness of screening practices
Behaviours	Screening history, screening behaviour(s)
Beliefs	Faith of cure, fate, perceived barriers, perceived benefits, perceived risk, perceived susceptibility, severity of cancer
Barriers	Embarrassment, lack of insurance, cost, language barriers, transportation, worry
Facilitators	Prior screening, good physician experience, social support, provider recommendation

## Data Availability

No new data were created or analyzed in this study. Data sharing is not applicable to this article.

## Supplementary Materials

The following supporting information can be downloaded at: https://www.mdpi.com/article/10.3390/curroncol30110715/s1, File S1: Questionnaire document that includes items asked in each Section; Table S1: Indigenous-specific Articles on Cancer Screening included from Scoping Review [30,35,38,39,40,41,45,46,47,48,49,50,51,52].
