# Supplementary material for: Creation of a Métis-Specific Instrument for Cancer Screening: A Scoping Review of Cancer-Screening Programs and Instruments
Source: Curr Oncol. 2023 Nov 9;30(11):9849–59. doi: 10.3390/curroncol30110715 (PMC10670396; doi:10.3390/curroncol30110715)
Supplement: Supplementary file 1 [file curroncol-30-00715-s001.zip › File S1. MNA Cancer Screening Questionnaire_Oct 18.pdf]

File S1. MNA Cancer Screening Questionnaire – ALL PARTICIPANTS

Creation of a Métis-specific instrument for cancer-screening: a scoping review of cancer-screening programs and instruments

Journal of Community Health: The Publication for Health Promotion and Disease Prevention

Angeline Letendre[1], Momtafin Khan[2], Reagan Bartel[3], Bonnie Chiang[4], Ashton James[3], Brittany Shewchuk[2], June Kima[3], Meghan Macphail[5], Marcus Vaska[6], Huiming Yang[4,5], Karen Kopciuk[2,4,5,7,*]

[1] Public Health Evidence & Innovation, Provincial, Population and Public Health, Alberta Health Services, Edmonton, AB, Canada

[2] Cancer Epidemiology and Prevention Research, Cancer Care Alberta, Alberta Health Services,

Calgary, AB, Canada

[3] Métis Nation of Alberta, Edmonton, AB, Canada

[4] Screening Programs, Alberta Health Services, Calgary, AB, Canada

[5] Department of Community Health Sciences, University of Calgary, Calgary, AB, Canada

[6] Knowledge Resource Service, Alberta Health Services, Calgary, AB, Canada

[7] Departments of Oncology and Mathematics and Statistics, University of Calgary, Calgary, AB, Canada

* Corresponding Author: Karen Kopciuk, ORCID iD 0000-0001-7499-186, email: kakopciu@ucalgary.ca

Informed Consent

Informed consent was required by each participant to access the survey questions. Consent details are summarized in the following text.

Participants were invited to participate in this research study because they are Métis with possible experience accessing cancer screening programs and initiatives in Alberta. The purpose of this study was described as helping to understand their experiences and perspectives with regards to cancer screening programs and initiatives. Detailed information about the objectives, methods, findings, dissemination of findings, voluntary nature of participation, and withdrawal were outlined in the consent forms. Potential risks included emotional distress while answering questions for which several helplines and their contact information were listed. Identifying information was not collected. Compensation was not given for this survey; however, participants could enter a draw for one of five $100 pre-paid Visa gift cards by clicking a link back to the MNA website. Participants were provided options for assistance to complete the survey online or if they required a language translation to Cree (from English.

The following questions were asked based on specific eligibility criteria for each cancer screening program with skip patterns not shown. All participants were asked to complete the demographic sections as well as barriers and facilitators to cancer screening.

Understanding

Do you understand that you have been asked to take part in a research study?

- ☐ Yes
- ☐ No

Do you understand why this study is being done?

- ☐ Yes
- ☐ No

Do you understand the potential benefits and risks/discomforts of participating?

- ☐ Yes
- ☐ No

Do you understand what you will be asked to do should you decide to take part in this study?

- ☐ Yes
- ☐ No

Do you understand that you are free to leave the study at any time, without having to give reason or without penalty?

- ☐ Yes

- ☐ No

Do you understand that we will be collecting information about you for use in this study only?

- ☐ Yes
- ☐ No

Do you feel that you had enough time and opportunity to consider the information provided to you by way of asking questions, having conversations with others and considering your options?

- ☐ Yes
- ☐ No

Translator/Interpreter Acknowledgement (If applicable)

This section is to be completed only if the participant requires the assistance of a qualified oral translator/interpreter. The interpreter must be impartial.

Do you require the help of a translator to complete this survey?

- ☐ Yes
- ☐ No

The informed consent discussion was accurately explained to and apparently understood by the research participant.

- ☐ Yes
- ☐ No

A slight translation of the consent document was provided by the interpreter as directed by the research staff conducting the consent process.

- ☐ Yes
- ☐ No

By clicking 'Yes' below, you are consenting to Yes participate in this survey. You are free to withdraw No at any time. Do you wish to continue to the survey?

- ☐ Yes
- ☐ No

SECTION A: Background and Cancer Screening History

Thank you for your interest in participating in our Cancer Screening Survey!

This survey contains questions where we will ask you about your background and cancer screening history, as well as your personal barriers and facilitators to cancer screening.

You do not need to complete the entire questionnaire and are free to withdraw from the survey at any time without impacting your ability to access health services or your relationship with the MNA.

QUESTION SOURCE KEY

Purple: MNA (Métis Nation of Alberta)

Red: CCHS 2020 (Canadian Community Health Survey)

Blue: CCKP-64 (The Cervical-Cancer-Knowledge-Prevention-64 questionnaire)

Orange: Indigenous Cancer Screening Scoping Review

Green: Project team

PART I: Background

1. Do you identify yourself as Métis?
   a. Yes
   b. No
   c. Unsure
   d. Prefer not to say

2. Are you a member of the Métis Nation of Alberta?
   a. Yes
   b. No
   c. Unsure
   d. Prefer not to say

3. What is your age in years?

   [SCALE FROM 0-100]

4. How would you describe yourself? (Note: This is required in order to determine your eligibility for cancer screening)
   a. Assigned female at birth
   b. Assigned male at birth
   c. Assigned male at birth and have been on feminizing therapy for 5+ years in total
   d. Assigned male at birth, have been on feminizing therapy for 5+ years in total, and have a cervix
   e. Assigned female at birth and have undergone top surgery (mastectomy), but have a cervix
   f. Assigned female at birth and have undergone top surgery (mastectomy) and hysterectomy (removal of uterus including cervix)
   g. Prefer not to say

PART II: Screening History

The following infographic summarizes the different screening programs that currently exist in Alberta for breast, cervical and colorectal cancer. The eligibility criteria are also included below, although it should be noted that the frequency of recommended screening may vary by individual and should be discussed with your healthcare provider.

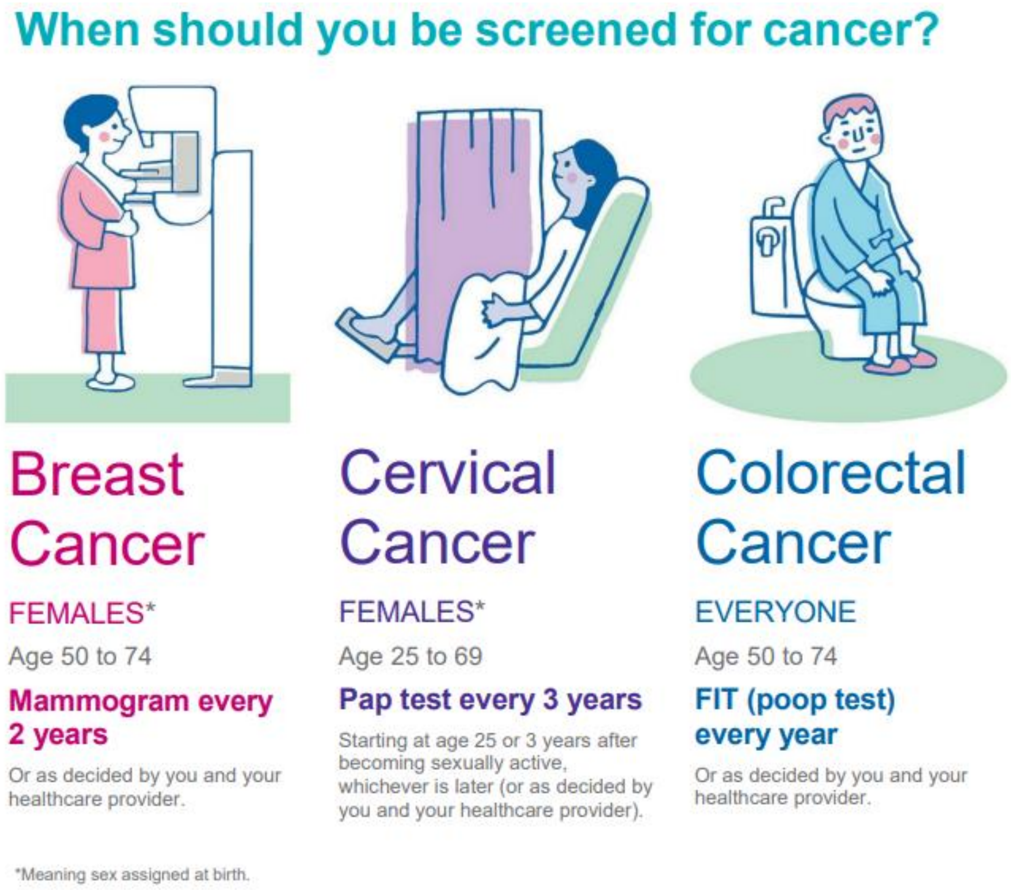

5. Have you been told by a doctor that you are eligible for cancer screening (due to age, family history, or other factors)?
   a. Yes
   b. No
   c. Unsure
   d. Prefer not to say

PART IIa: Breast Cancer Screening History

What is Breast Cancer Screening?

For most people, breast cancer screening begins when you are between the ages of 50 and 74 and is recommended every 2 years. However, if you have a personal or biological family history that puts you at higher risk of breast cancer, you may have to start screening between the ages of 25 and 40.

A 'screening mammogram' is an x-ray of the breast that is used to find early signs of cancer when there are no noticeable breast problems or symptoms. They can find abnormal changes in the breast that are too small to be noticed by you.

1. Have you ever had a Mammogram, that is, a breast x-ray?
   a. Yes
   b. No
   c. Unsure
   d. Prefer not to say

2. When was the last time you had a mammogram? (Note: we are just looking for you best guess)
   a. Less than 1 year ago
   b. 1 year to less than 2 years ago
   c. 2 to 3 years ago
   d. More than 3 years ago
   e. Not sure
   f. Prefer not to say

3. Why did you have your last mammogram (Select ALL that apply):
   - ☐ Family history of breast cancer
   - ☐ Part of regular checkup/routine screening
   - ☐ Age
   - ☐ Recommended by my doctor
   - ☐ Previously detected lump
   - ☐ On hormone replacement therapy
   - ☐ Breast problem
   - ☐ Prefer not to say
   - ☐ Other (please specify): ____________________

4. What are the reasons you may **not** have had a mammogram within the recommended timeframe? Select all that apply.
   - ☐ Lack of time
   - ☐ I did not think it was necessary
   - ☐ My doctor did not think it was necessary/ they never brought it up
   - ☐ I am worried about discomfort or safety

- ☐ I do not have a doctor
- ☐ I have had a bilateral mastectomy (both breasts were removed)
- ☐ I have had a mammogram within the past 3 years
- ☐ I am not eligible for breast cancer screening
- ☐ I did not know how to access screening
- ☐ Prefer not to say
- ☐ Other (please specify): __________

PART IIb: Cervical Cancer Screening History

What is Cervical Cancer Screening?

A Pap test (or Pap smear) is the main screening test for cervical cancer. Anyone between the ages of 25 to 69 with a cervix who has ever been sexually active, regardless of sexual orientation or gender identity, should consider getting regular Pap tests, even if they have been vaccinated against Human Papilloma Virus (HPV). In Alberta, eligible women typically receive a Pap test every 3 years.

Getting regular Pap tests is important to take care of your wellness. A Pap test looks for abnormal cells on your cervix that could lead to cancer before you have any signs or symptoms. Screening can even catch cancer early, when it is easiest to treat, despite you feeling healthy.

1. Have you ever had a Pap smear test?

a. Yes
b. No
c. Unsure
d. Prefer not to say

2. When was the last time you had a Pap smear test? (Note: we are just looking for your best guess)

a. 1 year or less
b. 1 to 3 years ago
c. 3 to 4 years ago
d. More than 4 years ago
e. Unsure
f. Prefer not to say

3. Why did you have your last Pap smear test (Select ALL that apply):

- ☐ Family history of cervical cancer
- ☐ Part of regular checkup/routine screening
- ☐ Age
- ☐ It was recommended by my doctor
- ☐ Previously detected abnormality
- ☐ Cervix problem
- ☐ Prefer not to say
- ☐ Other (please specify): ____________________

4. What are the reasons that you may **not** have had a Pap smear test [in the past 3 years](Select ALL that apply)?
- ☐ Lack of time
- ☐ I did not think it was necessary
- ☐ My doctor did not think it was necessary/ they never brought it up
- ☐ I am worried about discomfort or safety
- ☐ I don't have a doctor
- ☐ I have had a complete hysterectomy
- ☐ I do not have a cervix
- ☐ I have had a Pap smear within the past 3 years
- ☐ I am not eligible for cervical cancer screening
- ☐ I did not know how to access screening
- ☐ Prefer not to say
- ☐ Other (please explain): ____________

Human papillomavirus (HPV) is a common virus that affects most people at some point in their lifetime. Some types of HPV are considered "high-risk" and can cause abnormal cells to develop in the cervix that can lead to cervical cancer if left undetected.

One of the things you can do to protect yourself against HPV is to get the HPV vaccine before you become sexually active (sexual activity includes any skin-to-skin contact in the genital area). If you're already sexually active, you may still benefit from getting the vaccine. The vaccine can prevent 7 types of HPV that cause nearly 90% of all cases of cervical cancer.

5. Have you heard about the vaccine to prevent cervical cancer (HPV vaccine, Gardasil®)?
   a. Yes
   b. No
   c. Unsure
   d. Prefer not to say

6. Have you ever received the HPV vaccine?
   a. Yes
   b. No
   c. Unsure
   d. Prefer not to say

7. If you were eligible for the HPV vaccine, would you take it?
   a. Yes
   b. No
   c. Unsure
   d. Prefer not to say

8. If you feel comfortable doing so, please share why you may be hesitant to take the HPV vaccine? There are no right or wrong answers, we are simply looking for your opinion.

PART IIc: Colorectal Cancer Screening History

What is Colorectal Cancer Screening?

For most people, colorectal cancer screening starts at age 50 until you are 74. However, if you have a personal or family history that puts you at higher risk of colorectal cancer, you may have to start screening at age 40 or younger. In Alberta, eligible individuals are screened once a year, or as decided by you and your health care provider.

There are two main types of screening tests available. The type of screening you need to have will depend on your risk of developing colorectal cancer. Your risk is based on your biological family and personal history.

1) A fecal immunochemical test (FIT) Test (poop test) is a home poop test that looks for traces of blood in your poop that you cannot see that may be from polyps or cancer. You have a bowel movement and use a small plastic stick to collect a small sample of stool and put the stick into a collection container.

2) A colonoscopy is an exam in which a doctor inserts a flexible tube into the rectum to examine the entire colon to look for polyps or abnormal growths. A colonoscopy is done in a clinic or hospital. Before the procedure is done, you are usually given medication through a needle in your arm to make you sleepy.

1. Have you ever had a fecal immunochemical test (FIT) or poop test?
   a. Yes
   b. No
   c. Unsure
   d. Prefer not to say

2. When was the last time you had a poop test done (Note: we are just looking for you best guess)?
   a. Less than 1 year ago
   b. 1 to 2 years ago
   c. 2 to 3 years ago
   d. 3 or more years ago

e. Unsure
f. Prefer not to say

3. Why did you have your last poop test? (Select ALL that apply):
- ☐ Family history of colon or rectal cancer
- ☐ Part of regular check-up/ routine screening
- ☐ Age
- ☐ Signs or symptoms of a possible problem
- ☐ Follow-up of previous problem
- ☐ Prefer not to say
- ☐ Other (please specify): __________

4. What are the reasons that you may **not** have had a FIT or poop test in the past year [Select all that apply]?
   a. Lack of time
   b. I did not think it was necessary
   c. Doctor did not think it was necessary/ they never brought it up
   d. Feelings of fear or discomfort
   e. Don't have a doctor
   f. Have had a poop test or within the past year
   g. I am not eligible for colorectal cancer screening
   h. I did not know how to access screening
   i. Prefer not to say
   j. Other (please specify): ____________

5. Have you ever had a colonoscopy done?
   a. Yes
   b. No
   c. Unsure
   d. Prefer not to say

6. About how many times in total have you had a colonoscopy in your lifetime? (Note: we are just looking for your best guess)
   a. 1 colonoscopy
   b. 2 or more colonoscopies
   c. Unsure
   d. Prefer not to say

7. Why did you have your last colonoscopy exam (Select ALL that apply)?
- ☐ Family history of colon or rectal cancer
- ☐ Part of a regular checkup/ routine screening
- ☐ Age
- ☐ Signs or symptoms of a possible problem
- ☐ Follow-up of a previous problem
- ☐ Prefer not to say

- ☐ Other (Please specify): _____________

8. What are the reasons that you may **not** have had a colonoscopy in the past year [Select all that apply]?
   - ☐ Lack of time
   - ☐ I did not think it was necessary
   - ☐ Doctor did not think it was necessary/ they never brought it up
   - ☐ Feelings of fear or discomfort
   - ☐ Don't have a doctor
   - ☐ Have had a colonoscopy within the past year
   - ☐ I am not eligible for colorectal cancer screening
   - ☐ Other (please specify): ___________

SECTION B: Barriers and Facilitators to Screening

Indigenous peoples can experience barriers or facilitators to cancer screening. Screening **barriers** are factors that may prevent you from seeking or receiving screening, while **facilitators** are factors that may improve your access to screening or willingness to participate in screening programs. The presence of barriers can mean that cancer is not prevented when it could be or that cancer is not caught early when it is easiest to treat. Each person will experience barriers unique to their own life experiences. For example, trauma, past experiences of racism in healthcare, fear of screening results, and fear of not knowing what happens next if the results detect cancer.

The following questions are related to different barriers and facilitators that you may have experienced when it comes to cancer screening participation. Although not all of the barriers or facilitators mentioned below will be relevant to you, please answer to the best of your ability.

For each of the following factors please select a number indicating how important this factor is to you (1= not important, 5= very important) when considering participation in cancer screening:

**Access to health care and health care providers**					
Access to reliable information on screening	**1** Not important	**2** Somewhat unimportant	**3** Neutral	**4** Somewhat important	**5** Very important
Distance to nearest health facility	**1** Not important	**2**	**3** Neutral	**4**	**5** Very important
Access to permanent health facility	**1** Not important	**2**	**3** Neutral	**4**	**5** Very important
Transportation support (shuttles, vouchers)	**1** Not important	**2**	**3** Neutral	**4**	**5** Very important
Flexible appointment times (e.g., outside of business hours)	**1** Not important	**2**	**3** Neutral	**4**	**5** Very important

Acceptable staffing of clinics with health care providers	**1** Not important	**2**	**3** Neutral	**4**	**5** Very important
Short wait times for appointments	**1** Not important	**2**	**3** Neutral	**4**	**5** Very important
Access to a regular primary health care provider	**1** Not important	**2**	**3** Neutral	**4**	**5** Very important
Providing cancer screening test results in a timely manner	**1** Not important	**2**	**3** Neutral	**4**	**5** Very important
**Trust**					
Trust in the health care system	**1** Not important	**2**	**3** Neutral	**4**	**5** Very important
Trust in your health care provider (s)	**1** Not important	**2**	**3** Neutral	**4**	**5** Very important
Screening was recommended by a health care provider	**1** Not important	**2**	**3** Neutral	**4**	**5** Very important
Trust in screening technology (safety, accuracy)	**1** Not important	**2**	**3** Neutral	**4**	**5** Very important
**Respect**					
Availability of cultural supports	**1** Not important	**2**	**3** Neutral	**4**	**5** Very important
Presence of culturally competent staff	**1** Not important	**2**	**3** Neutral	**4**	**5** Very important
Acceptance of traditional approaches to medicine	**1** Not important	**2**	**3** Neutral	**4**	**5** Very important
Lack of discrimination and racism	**1** Not important	**2**	**3** Neutral	**4**	**5** Very important
Use of Indigenous languages	**1** Not important	**2**	**3** Neutral	**4**	**5** Very important
**Communication**					
Providing accurate information about cancer screening	**1** Not important	**2**	**3** Neutral	**4**	**5** Very important
Providing information of the risks and benefits of cancer screening	**1** Not important	**2**	**3** Neutral	**4**	**5** Very important
Clearly explaining the screening test or procedure	**1** Not important	**2**	**3** Neutral	**4**	**5** Very important
Use of appointment reminders	**1** Not important	**2**	**3** Neutral	**4**	**5** Very important
**Individual factors**					
Being employed or having income	**1** Not important	**2**	**3** Neutral	**4**	**5** Very important

Social support (family, friends)	**1** Not important	**2**	**3** Neutral	**4**	**5** Very important
Family history of cancer	**1** Not important	**2**	**3** Neutral	**4**	**5** Very important
Understanding of health information	**1** Not important	**2**	**3** Neutral	**4**	**5** Very important
Outside encouragement (e.g., from friends, family)	**1** Not important	**2**	**3** Neutral	**4**	**5** Very important
Belief that screening can detect cancer	**1** Not important	**2**	**3** Neutral	**4**	**5** Very important

a. Are there any factors not listed above that are important to you when it comes to your participation in cancer screening programs? If so, please explain:

SECTION C: Demographics

1. What is your highest level of completed education?
   a. Primary/elementary school or less
   b. Secondary/high school
   c. Red Seal/Trades certificate
   d. College or University degree
   e. Graduate or professional degree
   f. I have never been to school
   g. Prefer not to say

2. What is your current relationship status?
   a. Single
   b. Separated
   c. Divorced or widowed
   d. In a relationship/married, living apart
   e. In a relationship/married, living together
   f. Prefer not to say

3. Which of these describe your household income last year?
   a. Less than $49,999 per year
   b. $50,000 to $74,000 per year
   c. $75,000 to $100,000 per year
   d. Over $100,000 per year
   e. Prefer not to say

4. Thinking of the Métis Nation of Alberta regions, where is your regular place of residence located? Check all that apply:
   a. Region 1 – e.g., Lac La Biche
   b. Region 2 – e.g., Bonnyville
   c. Region 3 – e.g., Calgary
   d. Region 4 – e.g., Edmonton
   e. Region 5 – e.g., Slave Lake
   f. Region 6 – e.g., Peace River
   g. Unsure
   h. Prefer not to say

5. Do you regularly live *(check all that apply)*:
   a. In a city
   b. In a small town
   c. In a rural area
   d. In a remote area
   e. Prefer not to say

6. Currently, your housing situation is:
   a. Living in a home/apartment you own or rent
   b. Living in a home/apartment a family member owns or rents
   c. Living in a long-term care facility or group home
   d. Couch surfing, using a shelter, or rough sleeping
   e. Other:________
   f. Prefer not to say

7. What best describes your current employment status?
   a. In high school
   b. In University
   c. Retired
   d. Self-employed
   e. Part-time employment
   f. Full-time employment
   g. Homemaker/full-time parent
   h. Unemployed before the COVID-19 outbreak and seeking work
   i. Employed before the COVID-19 outbreak but laid off during the pandemic
   j. Prefer not to say

8. What is your current or most recent main area of occupation?
   a. Professional (health, physical/earth science/engineering professionals, teacher/university professor, business/sales and marketing professional, software developer, legal, law enforcement, clergy, author, journalist, performing arts)
   b. Manager (chief executive, administrative manager, production and sales, hospitality, and retail)

c. Technical or associate professional (in field of health, engineering, business, legal, social, or information/communication)
d. Clerical support worker (office clerk, secretary, customer service clerks)
e. Service and sales worker (travel agent, cook, hair dresser/barber, retail sales, cashier, personal care worker)
f. Skilled agricultural, forestry, and fishery worker
g. Craft and related trades worker (builders, machinists, electricians, printing, food processing)
h. Plant and machine operator and assembler (includes truck drivers)
i. Elementary occupations (cleaner, helper, agricultural laborer, transport laborer, street vendor, refuse worker)
j. Fishing or trapping
k. Home-maker (stay at home parent)
l. Prefer not to say

The survey is now complete. Thank you for participating! If you would like to be entered into a draw to win a $100 prepaid Visa card, please click on this link to enter on the MNA website. Note: your contact information will be kept private and cannot be linked to your survey responses.

If you are interested in learning more about cancer screening programs in Alberta, please click the link below:

Alberta Native Friendship Centres – Screening Pathways:

- **Breast cancer:** Breast-Cancer-Screening-Pathway.pdf (anfca.com)
- **Cervical cancer:** Cervical-Cancer-Screening-Pathway.pdf (anfca.com)
- **Colorectal cancer:** Colorectal-Cancer-Screening-Pathway.pdf (anfca.com)

Healthier Together Cancer Screening Information: https://www.healthiertogether.ca/living-healthy/get-screened/

If you are experiencing any distress, please contact the **MNA Supports and Services Navigator (780-455-2200, ext 403).**

For additional support, please visit any of the following resources below:

- Métis Nation of Alberta Wellness Program, a partnership with Alberta Blue Cross and Homewood Health offers mental health counselling to Métis Nation of Alberta citizens 24/7, free of charge. 1-844-729-0261
- Hope for Wellness Help Line offers immediate mental health counselling and crisis intervention to all Indigenous peoples across Canada 24/7. Visit them online or call 1-855-242-3310
- Health Link: 811
- Mental Health Helpline: 1-877-303-2642
- Provincial 211 (provides referrals for community, government, and social services)
