# Supplementary material for: Creation of a Métis-Specific Instrument for Cancer Screening: A Scoping Review of Cancer-Screening Programs and Instruments
Source: Curr Oncol. 2023 Nov 9;30(11):9849–59. doi: 10.3390/curroncol30110715 (PMC10670396; doi:10.3390/curroncol30110715)
Supplement: Supplementary file 1 [file curroncol-30-00715-s001.zip › Table S1.pdf]

Table S1. Indigenous-specific Articles on Cancer Screening included from the Scoping Review

Author (Year)	Study Design	N	Age (Years)	Target Population	Region	Cancer Screening (Breast/Cervical /Colorectal)	Instrument Included	Validated
Cerigo et al., 2011 [30]	Cross-Sectional	175 females	18-69	Inuit	Canada (Nunavik, Quebec)	Cervical	Yes	Yes
Smith et al., 2004 [35]	Community-based participatory research model	100 women	18+	Apsáalooke	United States (Crow Indian Reservation)	Cervical	Yes	Yes
Ndikum-Moffor et al., 2013 [38]	Qualitative	53 females	40+	American Indian/ Alaskan Native	United States	Breast	Yes	No
Wampler et al., 2006 [39]	Case-Control	271 females	≤41	American Indian (Northern Plains)	United States (Northern Plains Indian Reservation)	Breast	Yes	Yes
Brown et al., 2011 [40]	Qualitative	250 females	18+	Hopi	United States (Hopi Reservation)	Breast	Yes	Yes
Brown et al., 2015 [41]	Qualitative	252 females	NA	Hopi	United States (Hopi Reservation)	Breast, cervical, colorectal	No	No

Chow et al., 2020 [45]	Qualitative	841 total (685 females, 156 males)	Not specified but noted 21-74 age group included in screening programs	First Nations	Canada (Northwestern Ontario)	Breast, cervical, colorectal	Yes	No
Engelman al., 2010 [46]	Mixed Methods	12 focus groups, 8 to 12 females per group	40+	American Indian/ Alaskan Native	United States	Breast	No	Yes
Haverkamp et al., 2011 [47]	Qualitative	227	NA	Indian Health Service (IHS) and tribal providers	United States	Colorectal	Yes	No
Moreland et al., 2006 [48]	Cross-Sectional	116 females (48 Hispanic, 68 Native American)	18-89	Hispanic, Native American	United States	Cervical	Yes	No
Orians et al., 2004 [49]	Multiple-site Case Study	141 females	NA	Arctic Slope Native Association, Cherokee Nation, Cheyenne River Sioux Tribe, Southcentral Foundation, South Puget Intertribal	United States (Alaska, Oklahoma, South Dakota, Washington)	Cervical, breast	No	No

				Planning Agency				
Sanderson et al., 2011 [50]	Cross-Sectional	285 total (228 women, 55 men)	18-79	Navajo	United States	Colorectal	Yes	No
Solomon et al., 1999 [51]	Cross-Sectional	199 Women	18-40	American Indian	United States	Cervical	Yes	Yes
Soonhee et al., 2016 [52]	Cross-Sectional	181 total (97 female, 84 male)	50+	American Indian	United States	Colorectal	Yes	No

References

30. Cerigo H, Macdonald ME, Franco EL, Brassard P. Awareness and knowledge about human papillomavirus among Inuit women in Nunavik, Quebec. J Community Health. 2011;36(1):56-62. 10.1007/s10900-010-9278-x.

35. Smith A, Christopher S, McCormick AK. Development and implementation of a culturally sensitive cervical health survey: a community-based participatory approach. Women Health. 2004;40(2):67-86. https://doi.org/10.1300/J013v40n02_05.

38. Ndikum-Moffor FM, Braiuca S, Daley CM, Gajewski BJ, Engelman KK. Assessment of mammography experiences and satisfaction among American Indian/Alaska Native women. Womens Health Issues. 2013;23(6):e395-402. 10.1016/j.whi.2013.08.003.

39. Wampler NS, Ryschon T, Manson SM, Buchwald D. Knowledge, Attitudes, and Beliefs Regarding Breast Cancer Among American Indian Women From the Northern Plains. Journal of Applied Gerontology. 2006;25(1_suppl):44S-59S. 10.1177/0733464805282729.

40. Brown SR, Nuno T, Joshweseoma L, Begay RC, Goodluck C, Harris RB. Impact of a community-based breast cancer screening program on Hopi women. Preventive medicine. 2011;52(5):390-3. 10.1016/j.ypmed.2011.02.012.

41. Brown SR, Joshweseoma L, Saboda K, Sanderson P, Ami D, Harris R. Cancer screening on the Hopi reservation: A model for success in a Native American community. Journal of Community Health: The Publication for Health Promotion and Disease Prevention. 2015;40(6):1165-72. http://dx.doi.org/10.1007/s10900-015-0043-z.

45. Chow S, Bale S, Sky F, Wesley S, Beach L, Hyett S, Heiskanen T, Gillis K-J, Paroschy Harris C. The Wequedong Lodge Cancer Screening Program: implementation of an opportunistic cancer screening pilot program for residents of rural and remote Indigenous communities in Northwestern Ontario, Canada. Rural and remote health. 2020;20(1):5576. 10.22605/RRH5576.

46. Engelman KK, Daley CM, Gajewski BJ, Ndikum-Moffor F, Faseru B, Braiuca S, Joseph S, Ellerbeck EF, Greiner KA. An assessment of American Indian women's mammography experiences. BMC Womens Health. 2010;10:34. 10.1186/1472-6874-10-34.

47. Haverkamp D, Perdue DG, Espey D, Cobb N. A survey of Indian Health Service and tribal health providers' colorectal cancer screening knowledge, perceptions, and practices. J Health Care Poor Underserved. 2011;22(1):243-57. 10.1353/hpu.2011.0014.

48. Moreland S, Engelman K, Greiner KA, Mayo MS. Papanicolaou testing among Native American and Hispanic populations. Ethn Dis. 2006;16(1):223-7.

49. Orians CE, Erb J, Kenyon KL, Lantz PM, Liebow EB, Joe JR, Burhansstipanov L. Public education strategies for delivering breast and cervical cancer screening in American Indian and Alaska Native populations. Journal of public health management and practice : JPHMP. 2004;10(1):46-53. 10.1097/00124784-200401000-00009.

50. Sanderson PR, Weinstein N, Teufel-Shone N, Martínez ME. Assessing colorectal cancer screening knowledge at tribal fairs. Prev Chronic Dis. 2011;8(1):A16.

51. Solomon TG, Gottlieb NH. Measures of American Indian traditionality and its relationship to cervical cancer screening. Health care for women international. 1999;20(5):493-504.

52. Soonhee R, Burnette CE, Kyoung Hag L, Yeon-Shim L, Goins RT. Correlates of Receipt of Colorectal Cancer Screening among American Indians in the Northern Plains. Social Work Research. 2016;40(2):95-104. 10.1093/swr/svw006.
